# CancerPDF: A repository of cancer-associated peptidome found in human biofluids

**DOI:** 10.1038/s41598-017-01633-3

**Published:** 2017-05-04

**Authors:** Sherry Bhalla, Ruchi Verma, Harpreet Kaur, Rajesh Kumar, Salman Sadullah Usmani, Suresh Sharma, Gajendra P. S. Raghava

**Affiliations:** 1 Bioinformatics Centre, CSIR-Institute of Microbial Technology, Sector 39A, Chandigarh, 160036 India; 2 Centre for Systems Biology and Bioinformatics, Panjab University, Sector 14, Chandigarh, 160014 India

## Abstract

CancerPDF (Cancer Peptidome Database of bioFluids) is a comprehensive database of endogenous peptides detected in the human biofluids. The peptidome patterns reflect the synthesis, processing and degradation of proteins in the tissue environment and therefore can act as a gold mine to probe the peptide-based cancer biomarkers. Although an extensive data on cancer peptidome has been generated in the recent years, lack of a comprehensive resource restrains the facility to query the growing community knowledge. We have developed the cancer peptidome resource named CancerPDF, to collect and compile all the endogenous peptides isolated from human biofluids in various cancer profiling studies. CancerPDF has 14,367 entries with 9,692 unique peptide sequences corresponding to 2,230 unique precursor proteins from 56 high-throughput studies for ~27 cancer conditions. We have provided an interactive interface to query the endogenous peptides along with the primary information such as m/z, precursor protein, the type of cancer and its regulation status in cancer. To add-on, many web-based tools have been incorporated, which comprise of search, browse and similarity identification modules. We consider that the CancerPDF will be an invaluable resource to unwind the potential of peptidome-based cancer biomarkers. The CancerPDF is available at the web address http://crdd.osdd.net/raghava/cancerpdf/.

## Introduction

Cancer is considered as the major public health concern worldwide and the second most health hazardous disease, causing deaths in the United States. Globally 14 million new cases and 8.2 million cancer-related deaths have been reported in 2012^1^. In 2017, there is an approximation of 63,990 new cases and 14,400 deaths from cancer in the United States^2^. Although the rate of survival has increased over the years, still it is meager. The lack of diagnosis at an early stage is one of the major hurdles in treating the cancer patients^3^. Cancer detection is often skewed due to the lack of accurate and non-invasive markers. Due to advances in genomics and proteomics, the probability to detect the cancer at an early stage has improved using peptide-based biomarkers^4^. In recent years, the peptide-based biomarkers have emerged as diagnostic tools in several foodborne diseases^5^, arthritis^6^, inflammatory disease^7^ as well as cancer^8, 9^. Thus it is imperative to understand the mechanism of action and processing of peptides in mammalian biofluids. Following are the few examples of peptide-based biomarkers; i) Insulin and C-peptide are used in case of diabetes^10^, ii) Calcitonin, and collagen fragments in case of osteoporosis^11–13^, iii) Pro-gastrin-releasing peptide for small cell lung carcinoma^14^, iv) β-amyloid 1–42 for Alzheimer’s disease^15^ and v) angiotensin II for hypertension^16^.

In past, large number of peptide repositories and computational resources have been developed to explore full potential of peptides in medical sciences^17–19^. Pepbank is the generalized database of biologically relevant peptides containing nearly twenty thousand peptides obtained using text mining^20^. Some of the peptide databases like PeptideAtlas^21, 22^ and SwePep^23^ are specifically derived from mass spectrometry proteomics data. PeptideAtlas is one of the largest repositories of peptides identified from tandem mass spectrometry experiments collected from human, mouse, yeast and several other organisms. Similarly, SwePep database contains approximately four thousand endogenous peptides from different tissues originated from diverse species. Some databases like PeptideDB^24^, Endogenous Regulatory OligoPeptide knowledgebase^25, 26^ and BIOPEP database^27^ are specifically made to store naturally occurring bioactive peptides. Recently databases have been developed for maintaining peptides important for designing anticancer drugs^28^. The CancerPPD^28^ contains 3,491 anticancer peptides and 121 anticancer proteins with diverse origin. Similarly, the TumorHoPe^29^ database contains peptides that can recognize tumor tissues and tumor associated microenvironment.

Despite several databases have been developed to maintain different classes of peptides in the past, there is no dedicated repository of peptides (peptidome) released in the tumor microenviroment during cancer progression. Thus, there is a need to compile cancer-associated peptides or cancer-peptidome found in human biofluid^30^. Cancer-peptidome can act as a rich source of peptide biomarkers as it represents the various cellular and enzymatic processes happening in the tumor microenvironment. The peptide patterns generated by peptidomics study can aid in understanding the pathology of the disease^31^. The study of endogenous peptide patterns also hints the alterations in protease activity in cancer microenvironment, which deepens the pathophysiological awareness of the disease^32^. The circulating peptides in cancer patients have shown to classify patient subtypes providing a direct therapeutic approach to those individuals at an earlier stage, which is otherwise not detectable^33, 34^. There have been many high-throughput studies in which peptidome of the various biofluids like plasma, serum, blood, urine and their peptide content in cancer patients have been reported^35–37^. In this light, different groups have collected data regarding plasma proteome and cancer secretome and made attempts to develop resources such as Plasma Proteome Database [10.1093/nar/gkt1251] and Human Cancer Secretome Database [10.1093/database/bav051] compiling this information at the protein level.

To the best of authors’ knowledge, no attempts have been made to organize all the endogenous peptides, detected in various biofluids from different human cancers using clinical samples. A repertoire of these peptides will certainly be helpful for the scientific community in studying and discovering new peptide-based cancer biomarkers. In order to facilitate scientific community, we have developed a resource called CancerPDF. This database offers comprehensive information on naturally occurring peptides in the biofluids of cancer patients and their expression status as reported by the original studies. This structured information can be used for identification of cancer biomarkers from proteomics data of biofluids. This database integrates various web-based tools to facilitate users in extracting and analyzing data. In order to provide access from the wide range of devices (like Smartphones, iPads, Tablets), we have developed web interface using responsive web templates.

## Results

### Database statistics

**Figure 1:**
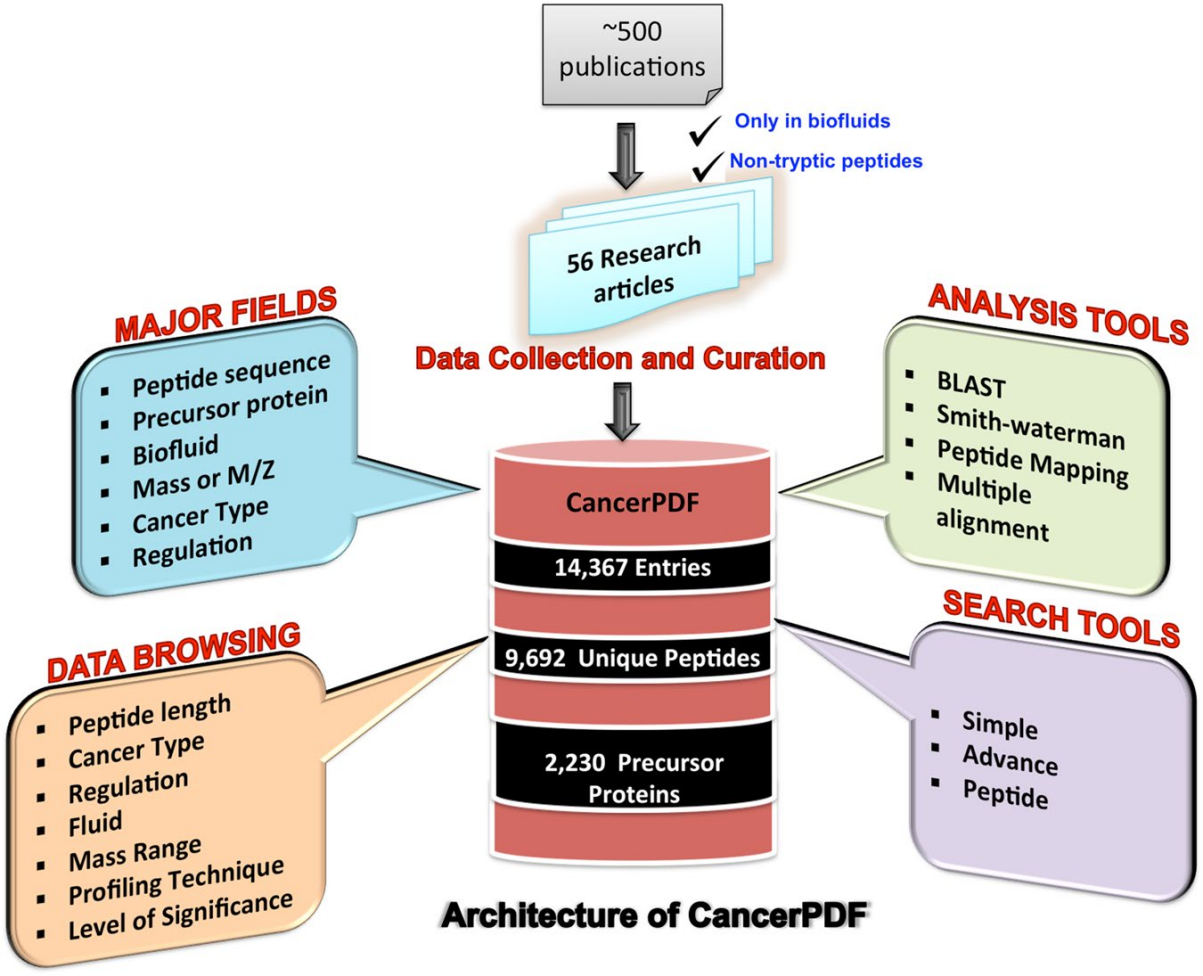
Architecture of CancerPDF database.

**Figure 2:**
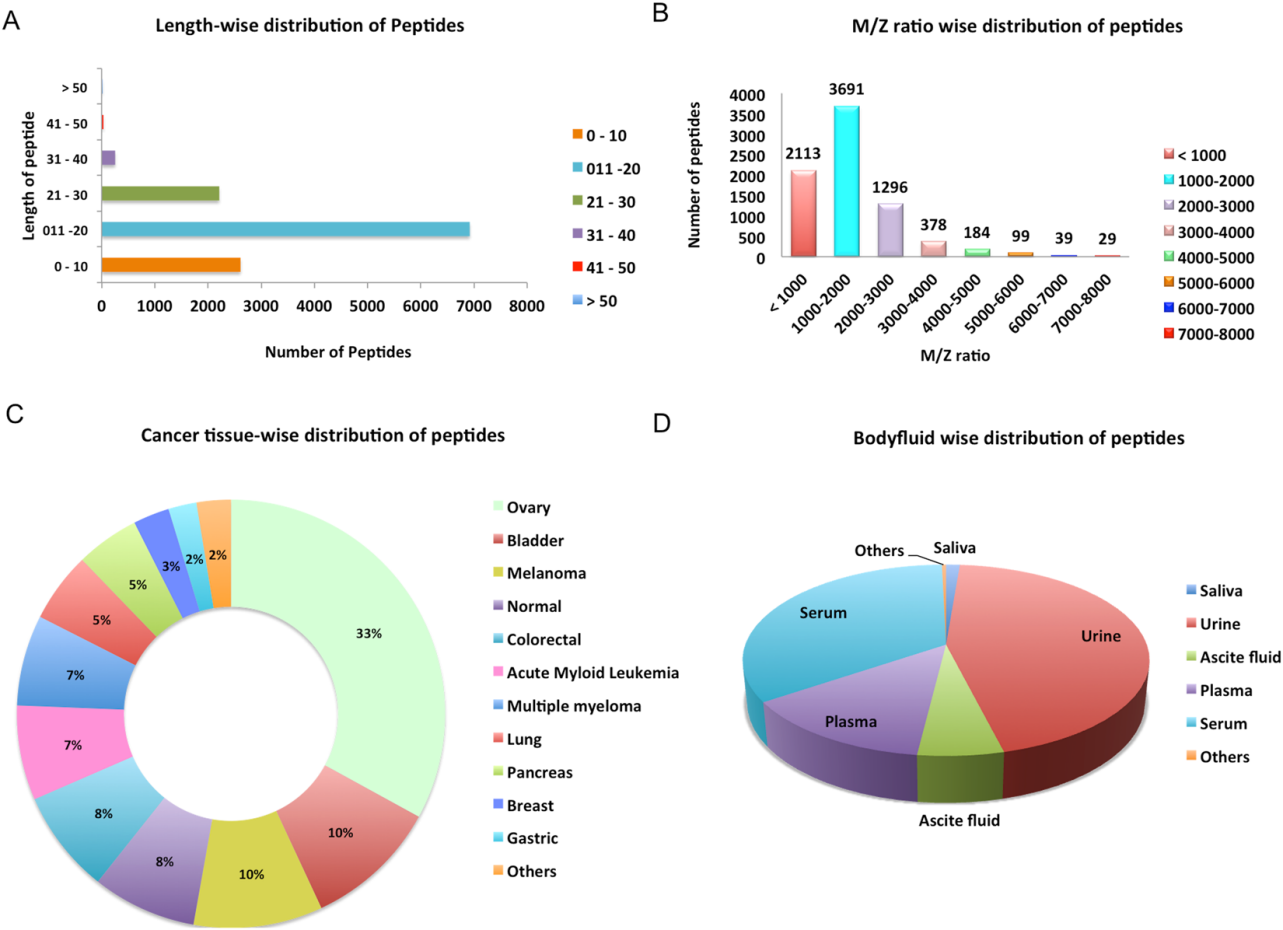
Distribution of peptides according to length (**A**), mass range (**B**), cancer tissue types (**C**) and biofluids (**D**) in CancerPDF database.

CancerPDF is a comprehensive resource of naturally occurring peptides found in biofluids using mass spectrometry. We have collected peptides, found only in the human biofluids from 56 studies which comprises of 14,367 entries corresponding to m/z values, out of which 9,692 entries have corresponding peptide sequences identified from 2,230 proteins (Fig. 1). The length of collected peptides in CancerPDF varies from 4 to 113 amino acid residues. Maximum peptides are in the range of 10–40 amino acid residues (Fig. 2A). The m/z values of endogenous peptides mostly varied from 300 to 14,000. Most of the peptides have mass in the range of 300 Da to 6,000 Da (Fig. 2B). The 56 studies encompassed nearly 27 different types of cancer conditions. The primary cancers according to tissues types are Ovary, Bladder, Melanoma, Colorectal and Multiple myeloma (Table 1 and Fig. 2C). Most of the peptides were derived from biofluids like urine, serum, plasma, ascites fluid, saliva and others with records corresponding to 5955, 4539, 2875, 777, 170 and 51 peptides respectively. Maximum studies were related to urine, plasma and serum, as they are most easy to obtain and non-invasive fluids which can be used to detect the cancer (Table 1 and Fig. 2D). These peptides are mainly profiled and identified using label-free mass spectrometry techniques such as LC/MS-MS and MALDI-TOF MS-MS. To assess the information about the precursor proteins from which these peptides are derived, we converted all the protein names to the UniProtKB entry names. CancerPDF peptides map to 2,230 unique UniProtKB entry names. The proteins for which the maximum numbers of peptides are found include FIBA_HUMAN, CO3_HUMAN, APOA1_HUMAN, CO1A1_HUMAN and A4_HUMAN (Table 2). Eight out of the top ten proteins with the highest number of identified peptides of CancerPDF are found to be differentially expressed in dbDEPC 2.0^38^, which is a database of differentially expressed proteins in cancer.

**Table 1:** Distribution of CancerPDF entries across key cancer types and major body fluids.

Biofluid	Serum	Plasma	Urine	Others	Total
Cancer					
Ovary	67	0	4368	777	5212
Bladder	80	0	1515	0	1595
Melanoma	1539	0	0	0	1539
Colorectal	1186	44	3	0	1233
Multiple myeloma	0	1083	0	0	1083
Lung	836	0	0	0	836
Pancreas	66	690	0	0	756
Breast	419	8	0	5	432
Gastric	335	0	0	1	336
Thyroid	103	0	0	0	103
Renal	36	0	62	0	98
Others	81	9	7	19	116
Total	4748	1834	5955	802	13339

**Table 2:** Top ten proteins with maximum numbers of reported peptides in CancerPDF.

**Uniprot**KB **entry name**	Number of Unique peptides	Number of Studies	Number of Cancer conditions
FIBA_HUMAN	727	25	21
CO3_HUMAN	296	16	15
APOA1_HUMAN	266	14	13
CO1A1_HUMAN	232	4	3
A4_HUMAN	223	12	12
A1AT_HUMAN	204	8	7
H4_HUMAN	200	20	16
APOA4_HUMAN	199	9	9
ITIH4_HUMAN	182	18	14
ALBU_HUMAN	168	11	11

Oxidation and hydroxylation are the most commonly occurring modifications in peptides, *i.e*. in 404 and 198 peptides, respectively. Another important aspect of these peptides is their differential regulation in various conditions like cancer versus normal. Wherever available, we have collected the information whether the peptides were differentially expressed, uniquely expressed, up-regulated and down-regulated in different conditions as reported in the corresponding studies. In this database, the peptides are reported to be differentially expressed in cancer versus healthy conditions, based on the level of significance (p-value < 0.05) reported in original study. CancerPDF comprises of 2,379 entries of differentially expressed peptides among diverse groups. Further there are 464 up-regulated, 355 down-regulated and nearly 5,152 uniquely expressed peptide peaks in various cancers. We have also specified the classification sensitivity, specificity and accuracy of the peptides biomarkers as reported in the respective studies (wherever possible) to provide an estimate of biomarker peptide efficiency.

### Implementation of web tools

To enable convenient data searching, various tools such as retrieval, browsing and analysis were integrated with CancerPDF.

### Search tools

We have implemented three different modules namely ‘Simple search’, ‘Peptide Search’ and ‘Advance search’ under the search option to provide a facility for the adequate data retrieval.

#### Simple search

This tool represents key data retrieval module from the CancerPDF. The keyword search can be executed by a user on the major fields of the database such as PubMed ID, Biofluids, Protein Name, Cancer Type, Regulation and Validation etc. Moreover, this module also allows the users to select various fields to be displayed for the result.

#### Peptide search

This tool offers a platform for searching a given peptide sequence against all peptide sequences available in CancerPDF. It searches for the exact match as well as substring matches in the database. Exact search option retrieves those peptides from the database, which have an identical amino acid sequence with the query peptide. While substring search option retrieves those peptides that contain the query peptide.

#### Advance search

This module assists the user to perform multiple structured query system options for the retrieval of the required information from the CancerPDF. By default, it performs four queries simultaneously, but a user can choose desired keyword search from any selected field. Besides this, advance search offers the user to apply standard logical operators (e.g. =, >, < and LIKE). Moreover, this module permits the user to integrate the output of different queries by utilizing operators like ‘AND and OR’. Additionally, the user can also add or remove the queries to be implemented.

### Browse tools

In CancerPDF, we have implemented browsing facility, which helps the user for convenient data navigation within the database in an orderly manner. In this module, a user can retrieve information on peptides by browsing nine different categories (i) Cancer Type, (ii) Fluid, (iii) Regulation, (iv) Precursor Protein, (v) Profiling Technique, (vi) Mass Range, (vii) Level of significance (p-value), (viii) Peptide Length and (ix) PubMed ID.

The ‘Cancer Type’ field facilitates the user to extract the information on peptides obtained from specific cancer conditions such as Lung cancer, Breast cancer, Prostate cancer etc. From the ‘Fluid’ category, the user is allowed to retrieve detailed information on the peptides isolated from a particular type of biofluid e.g. serum, plasma, urine and saliva. The ‘Regulation’ field offers the user to fetch the information on peptides that are up-regulated, down-regulated, differentially expressed in cancer condition as compared to healthy and peptides that are uniquely expressed in a specific type of cancer. In addition, by ‘Precursor Protein’ category, user can withdraw information on those peptides that are derived from a specific precursor protein such as Fibrinogen-alpha chain, Fibrinogen-beta chain and Complement component C3f etc. The ‘Profiling Technique’ option permits the user to extract information regarding the peptides that are profiled using different techniques such as MALDI-TOF, LC-MS etc. Furthermore, a user can also extract the information of peptides on the basis of their length by browsing ‘Mass Range’, ‘Level of significance (p-value)’, ‘Peptide Length’ and ‘PubMed ID’.

### Similarity

This module facilitates the user to perform various analyses such as sequence similarity, mapping and multiple sequence alignment by implementing different web-based tools i.e. Basic Local Alignment Search Tool (BLAST), Smith-Waterman, Multiple Sequence Alignment in CancerPDF database.

#### BLAST Search

This tool offers a user to execute a similarity-based search against CancerPDF database. Peptide sequences should be submitted in FASTA format and the user can choose different parameters such as weight matrix and an expectation value for the execution of BLAST search^39^.

#### Smith-Waterman Search

This algorithm executes similarity search against small peptides more efficiently using Smith-Waterman algorithm^40^. This module permits the user to search peptides in CancerPDF database similar to their query peptides. In this option, a user can submit simultaneously multiple peptide sequences in FASTA format.

#### Multiple Sequence Alignment (MSA)

This module offers the user to align their peptide sequences using ClustalW^41^ sequences along the peptides of CancerPDF Database. A user can perform batch submission in FASTA format in provided input box to get aligned sequences using MSA viewer^42^.

#### Peptide Mapping

This tool permits a user to map CancerPDF peptides over their peptide sequences. Under this module, the user can perform mapping using two options i.e. Sub search and Super search. In Sub search, query peptide is mapped across all the peptides in the CancerPDF, while Super search allows mapping of protein sequence against CancerPDF. The Super search module is useful to identify the local region of the query protein that is identical to peptides of CancerPDF.

### Comparison with other peptide and protein databases

CancerPDF database consists of endogenous peptides that are found in the biofluids of cancer patients. To understand the biological importance of these peptides, we compared the peptides using sequence-based similarity in CancerPDF with already existing peptide resources such as PeptideAtlas and immune epitope database and analysis resource (IEDB)^43^. We found numerous overlapping and exclusive peptides in CancerPDF as compared to these two resources (Supplementary Figure S1). Mapping peptides in CancerPDF with PeptideAtlas human build resulted in 2,007 common peptides. On comparing the CancerPDF with IEDB, 1,526 exact matches were found. Out of these, 1,301 were found to be MHC-I restricted peptides. This indicates the activation of the cell-mediated immune system during cancer progression; mediated via MHC-I restricted peptides. In literature, it is well known that cell-mediated immunity is triggered in the body during tumorigenesis, but becomes ineffective due to local suppressive factors at tumor sites^44–46^. This analysis shows that these peptides can be further explored for designing therapeutic vaccines against cancer based on MHC-I restricted peptides, due to their stability under cancerous conditions^47^.

Moreover, to understand the significance of proteins in our database, we have compared the precursor proteins of CancerPDF peptides with the database of differentially expressed proteins in cancers named dbDEPC 2.0^38^ and obtained 232 common UniProtKB entry names of proteins. This type of analysis indicated that the differentially expressed endogenous peptides reflect differentially expressed precursor proteins in cancer patients.

## Discussion

Peptidomics is an emerging field that deals with the comprehensive qualitative and quantitative analysis of peptides in biological samples^9^. During protein processing and degradation of other biological macromolecules, peptides are derived either from precursor protein or as degradation products. Therefore, subjecting to the physiological state of an organism, the amount of the peptide repertoire changes within body circulation. The pathological or diseased state has the direct effect on these peptide repertoires^48^. Detecting biomarkers in biofluids is one of the most extensive research interests in this era as it is the most non-invasive approach to uncover biomarker for various diseases^49^. The naturally occurring peptide patterns can be exploited to detect variations at the proteomics level of the tumor microenvironment^50^. The CancerPDF database provides the collection of endogenous peptides in the human biofluids and their precursor proteins that are found in the cancer peptidome profiling studies. As a comprehensive resource containing 14,367 entries, CancerPDF can aid in defining candidate peptide biomarkers derived from the biofluids in cancer. This database also stores the peptides that are differentially regulated and uniquely found in different types of cancer. CancerPDF can be a very important source to mine the peptides that are differentially regulated in specific type of cancer in different population cohorts and peptides that are differentially regulated across different types of cancer. Further analysis of a particular protein with its associated peptides in cancer will shed light on activation and deactivation of various proteolytic events specific to cancer. We foresee that CancerPDF will act as preliminary effort that will help in analyzing cancer peptidome associations and peptide-based cancer biomarker discovery.

## Utility of database

In the last decade several databases have been developed that maintain different type of information related to peptides and proteins. Thus it is essential to rationalize the need of another peptide database or the unique features of the CancerPDF. Some of the potential applications of the CancerPDF include.

### Screening of cancer biomarker

The CancerPDF includes the peptides and their precursor proteins that are differentially regulated in various cancer conditions. The user can easily identify number of differentially regulated peptides found in a particular type of cancer. The presence and absence of the differentially expressed peptides can be used as features for developing prediction models for discriminating cancer and healthy individuals. Thus CancerPDF is an important resource for developing biomarkers for the different types of cancer. These peptides are founds in bodyfluids that make them potential non-invasive biomarkers for detecting cancer.

This database can help in understanding the change in peptide content during developement of cancer (e.g., breast cancer). In order to demonstrate its application, we browsed the entries of the breast cancer. We obtained total 432 entries with 177 unique peptides that include 120 up-regulated, 28 down-regulated and 25 differentially expressed peptides (p-value < 0.05). It was observed that peptide sequence “MNFRPGVLSSRQLGLPGPPDVPDHAAYHPF”, has been found to be up-regulated in three different studies of breast cancer. This type of peptide is important while defining candidate peptide biomarkers as it has been found up-regulated in three independent population cohorts. So out of all reported peptides user can get these types of lead peptides to further confirm for biomarker potential.

### Peptide Library

A user can use the peptides in CancerPDF as the peptide library to directly search raw mass spectrometry cancer data to find the already known endogenous peptides in a particular sample. This will facilitate the researcher in identification of differentially regulated peptides in their sample that have been already annotated in previous studies.

### Pan-cancer analysis

CancerPDF offers the opportunity to search for those peptides that are differentially regulated across multiple types of cancers and also for those peptides that are differentially regulated in a specific cancer. One of the peptide sequences (SGEGDFLAEGGGVR) was found in 10 different studies and differentially regulated in 9 types of cancer (Table 3). These types of inferences can be crucial for further mining of peptide biomarkers for cancer.

**Table 3:** Top fifteen unique peptides associated with different cancers. Each number in the cell represents the number of studies associated with each cancer.

**Cancer→**	Prostate Cancer	Bladder Cancer	Breast Cancer	NSCLC	Lung adeno- carcinoma	Renal Cell carcinoma	Colorectal carcinoma	Metastatic thyroid carcinomas	Ovarian Cancer	ESCC	Cervical Cancer
**Sequence**											
ADSGEGDFLAEGGGVR	1	3	1	1	1	—	—	—	1	—	—
SGEGDFLAEGGGVR	1	2	1	1	1	1	1	1	1	—	—
RPPGFSPFR	1	2	3	—	—	—	1	—	—	—	—
DSGEGDFLAEGGGVR	1	2	1	1	1	1	1	1	—	—	—
SKITHRIHWESASLL	1	1	2	1	—	—	1	1	—	—	—
MNFRPGVLSSRQLGLPGPPDVPDHAAYHPF	1	1	5	—	—	—	—	—	—	1	—
SSKITHRIHWESASLL	1	1	2	1	—	—	—	1	—	—	—
RPPGFSPF	1	1	2	1	—	—	1	—	—	—	1
KITHRIHWESASLL	1	1	2	1	—	—	—	1	—	—	—
GEGDFLAEGGGVR	1	1	1	1	1	1	1	1	—	—	—
DEAGSEADHEGTHSTKRGHAKSRPV	1	3	4	—	—	—	—	—	—	—	—
SSSYSKQFTSSTSYNRGDSTFESKSYKM	1	1	2	—	1	—	—	1	—	—	1
NGFKSHALQLNNRQIR	1	1	2	—	—	—	1	—	—	—	1
DFLAEGGGVR	1	1	1	—	1	—	1	1	—	—	1
THRIHWESASLL	1	1	2	—	—	—	—	1	—	—	—

In summary, CancerPDF is an invaluable resource to the scientific community working in the area of peptide-based cancer diagnostics.

## Methods

### Data collection

We queried PubMed to obtain the research articles with the keywords “cancer [Title/Abstract] AND peptidome [Title/Abstract]” and “cancer [Title/Abstract]) AND endogenous [Title/Abstract] AND peptides [Title/Abstract]” and collected around 500 publications till September 2016. All research articles were curated manually to understand the type of information available in these articles. After reading all articles carefully, we kept articles for further processing that have information relevant to naturally occurring peptides extracted from body fluids. We excluded all those articles, for which peptides/peptidome were derived either using tryptic digestion, or from cell lines and tissues. We have also included publications that include peptidome of biofluids of normal individuals.

We manually retrieved information from selected articles regarding the sequence of peptides, precursor protein, their m/z value, mass (in Daltons or H^+^), charge, modification, profiling techniques, peptide identification technique, quantification techniques, their regulation, type of cancer, fluid sample from which peptides were extracted, and validation etc.

### Architecture and interface of database

CancerPDF is assembled employing Apache HTTP Server on Red Hat Linux system. A responsive web template is used as the web interface for the front end of this database. Thus web interface is compatible to the wide range of modern devices that includes Mobile, Tablet, Ipad, iMac and Desktop. The front end of the database is developed using HTML5, CSS3, PHP (version 5.2.14) and JavaScript (version 1.7). To manage the data efficiently, we used an object-relational database management system (RDBMS) MySQL at the back end. CancerPDF has numerous web-based tools to compile, explore and retrieve the information from the database.

### Organization of database

In CancerPDF, data is categorized into primary and secondary information. The primary information, procured from the research articles was arranged into defined categories which include (i) Peptide: its sequence, length and modification; (ii) Precursor protein: Protein name, as given in research article and UniProtKB entry name retrieved using *bioDBnet* tool^51^ and DAVID^52^; (iii) Physical properties of Peptide: m/z ratio, Mass (H^+^), Mass (in Daltons) and charge; (iv) Cancer aspects: Type of cancer, Number of cancer patients and Regulation status of peptide in cancer condition; (v) Biofluid from which peptide was isolated; (vi) Statistics of peptide identification: p-value and false discovery rate (FDR); (vii) Performance Measures: validation, sensitivity, specificity and accuracy; and (viii) Pubmed ID of research article from which information was extracted. In addition to primary information, in the secondary information category, each peptide is linked to IEDB and Peptide Atlas database wherever available.

## Availability

CancerPDF can be accessed freely at http://crdd.osdd.net/raghava/cancerpdf/.

## Electronic supplementary material

Supplementary Information
