# Supplementary material for: CancerPDF: A repository of cancer-associated peptidome found in human biofluids
Source: Sci Rep. 2017 May 4;7:1511. doi: 10.1038/s41598-017-01633-3 (PMC5431423; doi:10.1038/s41598-017-01633-3)
Supplement: Supplementary file 1 — Supplementary Information [file 41598_2017_1633_MOESM1_ESM.doc]

**Supplementary Information File**

**CancerPDF: A repository of cancer-associated peptidome found in human biofluids**

Sherry Bhalla^1*^, Ruchi Verma^1*^, Harpreet Kaur^1*^, Rajesh Kumar^1^, Salman Sadullah Usmani^1^, Suresh Sharma^2^, Gajendra P.S. Raghava^#1^

^1^Bioinformatics Centre, CSIR-Institute of Microbial Technology, Sector 39A, Chandigarh-160036, India

^2^Centre for Systems Biology and Bioinformatics, Panjab University, Sector 14, Chandigarh-160014, India

*Joint First Authors

**^#^Address for correspondence**

G.P.S. Raghava

Email: [raghava@imtech.res.in](mailto:raghava@imtech.res.in)

Web: <http://www.imtech.res.in/raghava/>

Phone: +91-172-2690557

Fax: +91-172-2690632

**
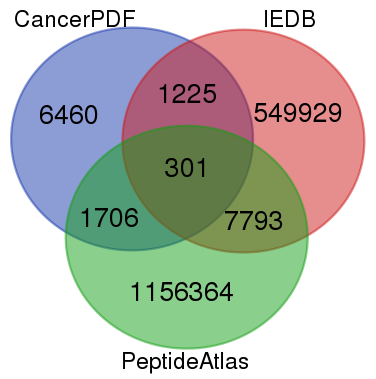
**

**Figure S1: Venn diagram showing comparison of CancerPDF with IEDB and PeptideAtlas.**
